# Prevalence of left ventricular thrombus formation after mitral valve edge-to-edge repair

**DOI:** 10.1038/s41598-022-12944-5

**Published:** 2022-05-31

**Authors:** Tobias Tichelbäcker, Maria Isabel Körber, Victor Mauri, Christos Iliadis, Clemens Metze, Christoph Adler, Stephan Baldus, Volker Rudolph, Marcel Halbach, Roman Pfister, Henrik ten Freyhaus

**Affiliations:** 1 Department of Cardiology, Heart Center of the University of Cologne, Kerpener Str. 62, 50939 Cologne, Germany; 2 Department of General and Interventional Cardiology, Heart and Diabetes Center North Rhine-Westphalia, Ruhr University Bochum, Bad Oeynhausen, Germany; 3 Cologne Cardiovascular Research Center (CCRC), University of Cologne, Cologne, Germany

**Keywords:** Cardiology, Interventional cardiology

## Abstract

The prevalence of left ventricular (LV) thrombus formation following percutaneous mitral valve edge-to-edge repair (TMVR) with the MitraClip system is unclear. Decreased total stroke volume and perfusion of the LV apex after mitral valve repair may facilitate thrombus formation especially in the context of reduced LV function. LV thrombus may cause disabling stroke or other thromboembolic events in this elderly and multimorbid patient cohort. Analyses of the prevalence of and risk factors for left ventricular thrombus formation in patients treated with the MitraClip system due to severe mitral valve regurgitation. All discharge and follow-up transthoracic echocardiographic examinations up to 6 months of 453 consecutive patients treated with the MitraClip system were screened for the presence of LV thrombus. Prevalence of LV thrombus formation was 1.1% (5/453). Importantly, LV thrombi were exclusively found in patients with severely depressed left ventricular systolic function (LV-EF < 30%), comprising a prevalence of 4.4% in this subgroup (5/113). Importantly, two of these patients were under active DOAC therapy with Rivaroxaban and Apixaban, respectively. Apart from LV-EF, we did not identify other factors that might have facilitated LV thrombus formation. LV thrombus formation following percutaneous mitral valve repair occurred exclusively in patients with severely depressed LV-EF. As two patients developed LV thrombus despite of DOAC therapy, anticoagulation with a Vitamin K antagonist should be considered in patients with an indication for oral anticoagulation following TMVR.

## Introduction

LV thrombus formation in patients with heart failure with reduced left ventricular (LV) ejection fraction (HFrEF), especially in the context of a dilated left ventricle, is a known complication with a prevalence up to 19% in this patient subgroup^1–3^. The presence of left ventricular thrombus is associated with a high risk of MACE and mortality^4^. Optimal therapy of left ventricular thrombus is still under debate. Whereas the use of Vitamin K antagonists is first-line therapy in most centers, small studies and case reports have demonstrated that direct oral anticoagulation (DOAC) therapy might be an alternative^5,6^. Percutaneous mitral valve therapy has become a valuable treatment option in patients with HFrEF and severe, mainly functional, mitral regurgitation (MR)^7^. Consequently, current guidelines advocate edge-to-edge repair of the mitral valve (TMVR) in selected patients^8^. This therapeutic approach leads to alteration of LV inflow through the newly created double or triple orifice and may interfere with the laminar perfusion of the LV apex, which is already impaired in the context of reduced left ventricular systolic function^9^. Based on these considerations, the formation of an apical LV thrombus may be facilitated after edge-to-edge therapy.

So far, only one case series with three patients has provided some insight regarding the development of LV thrombus formation in patients treated with the MitraClip system (MC)^10^. In this study, LV thrombus formation was found in approximately 20% of patients with severely depressed LV ejection fraction (in this study not guideline-conform defined as LV-EF < 20%). Thus, prevalence of LV thrombus formation in the context of mitral valve edge-to-edge therapy is unclear. Aim of this study was to evaluate the prevalence of LV thrombus formation following mitral valve edge-to-edge therapy at a high-volume center in Germany, to identify risk factors and to assess the effectiveness of the post-procedure anticoagulation regimens.

## Methods

We conducted a single center observational retrospective all-comers study of all consecutive patients treated with the MitraClip system between 11/2010 and 10/2017. All patients were discussed in a multi-disciplinary heart team prior to the procedure.

Patients were screened for the presence of LV thrombus in post-procedure transthoracic echocardiography (TTE) or early post interventional TTE examinations (up to six months post-procedure). If a LV thrombus was found, pre-procedure TTE were screened to exclude patients with preexisting LV thrombus. If a structure potentially representing a thrombus was found but the diagnosis was uncertain, or if left ventricular images were suboptimal, contrast echocardiography was performed to differentiate between thrombus and trabeculation according to current guidelines^11^. In our center, every patient was invited for outpatient visits including echocardiography after one, six- and twelve-months post-procedure.

Baseline characteristics, procedural measurements, as well as echocardiographic data prior and after MitraClip procedure were collected. These included LV dimensions, LV ejection fraction, mitral valve opening area, grade of mitral regurgitation, mitral valve gradient and sphericity index. Echocardiograms were obtained in the left lateral decubitus position under continuous ECG recording with a Philips IE 33 or a GE Vivid E95.

Routine dual antiplatelet therapy (DAPT) with aspirin and clopidogrel was administered following MitraClip procedure for one month and aspirin monotherapy for an additional 5 months. In patients with an indication for oral anticoagulation, their initial anticoagulation therapy without any supplementary medication was continued.

This is a retrospective analysis of a cohort that was approved by the local ethic committee (Ethic committee University of Cologne; No 13-019) and was conducted according to ICH-GCP standards and the declaration of Helsinki. The need for a written informed consent was waived by our local ethic committee (Ethic committee University of Cologne; No 13-019) due to the retrospective character of the study. The datasets used and analyzed during the current study are available from the first author upon request.

All data are expressed as mean values ± SD (or median, if indicated as such). Statistical analyses were performed using Fisher´s exact test. Statistical significance was defined as *p* < 0.05. Figures were created with BioRender.com.

## Results

### Baseline characteristics of the cohort

453 patients were included in the analyses (Table 1). Median age was 79.1 with a slight male predominance (260/453 corresponding to 57.4% male). 262 of the 453 patients had myocardial disease, 153 of ischemic origin and 109 non-ischemic. 113 patients had severely depressed left ventricular systolic function (LV-EF < 30%). Renal function in the cohort was mostly impaired (mean GFR 46 ± 22 ml/min) and co-morbidities such as diabetes mellitus, COPD and history of stroke were frequently observed. Mitral regurgitation prior to intervention was severe (grade 3–4) with the majority of patients presenting with grade 4 regurgitation (65.5%).

**Table 1:** Baseline characteristics of the MitraClip cohort.

Baseline charateristics (n = 453)	
Age (median)	79.1
Sex male	260/453 (57.4%)
BMI	25.5 ± 4.9
ICM	153/453 (33.8%)
DCM	109/453 (24.6%)
**Mitral regurgitation**	
Grad 3	156/453 (34.4%)
Grad 4	297/453 (65.6%)
GFR (ml/min) (mean ± SD)	46 ± 22
COPD	81/453 (17.9%)
History of stroke	63/453 (13.9%)
Atrial fibrillation	274/453 (60.5%)
Diabetes mellitus	118/453 (26%)
Art. hypertension	325/453 (71.7%)
Coronary heart disease	261/453 (57.6%)
History of CABG	100/453 (22.1%)
BNP (ng/l) (mean ± SD)	5683 ± 9683
Primary mitral regurgitation	178/453 (39.3%)
Secondary mitral regurgitation	134/453 (29.6%)
Mixed aetiology	161/453 (35.5%)

### Prevalence of LV thrombus formation after TMVR

We identified LV thrombus formation in five patients, corresponding to a prevalence of 1.1% (5/453). All patients with LV thrombus diagnosis had severely depressed LV-EF, no LV thrombi were identified in the subgroup of patients with LV-EF ≥ 30%The prevalence of diagnosed LV thrombus in the cohort with LV-EF < 30% was 4.4% (5 of 113 patients) and obviously statistically significantly higher than in patients with EF ≥ 30% (p < 0.0001). Consequently, we carried out further analyses with the two subgroups divided by a LV-EF of 30%. Baseline characteristics of the subgroups is given in Table 2. Patients with severely depressed LV-EF more likely suffered from functional MR and numerically had higher BNP levels. Furthermore, there were no obvious differences between groups.

**Table 2:** Comparison of MC patients and HFrEF MC patients.

Baseline charateristics	LVEF ≥ 30% (n = 340)	LVEF < 30% (n = 113)
Age (median)	79.2	79.2
Sex male	180/340 (52.9%)	80/113 (70.8%)
BMI	25.6 ± 4.8	25.2 ± 4.6
ICM	111/340 (32.6%)	42/113 (37.2%)
DCM	78/340 (22.9%)	31/113 (27.4%)
**Mitral regurgitation**		
Grad 3	121/340 (35.6%)	35/113 (31%)
Grad 4	219/340 (64.4%)	78/113 (69%)
GFR (ml/min) (mean ± SD)	45.6 ± 22	47.1 ± 21.9
COPD	63/340 (18.5%)	18/113 (15.9%)
History of stroke	44/340 (12.9%)	19/113 (16.8%)
Atrial fibrillation	218 (64.1%)	56/113 (49.6%)
Diabetes mellitus	82/340 (24.1%)	36/113 (31.9%)
Art. hypertension	246/340 (72.4%)	79/113 (70%)
Coronary heart disease	197/340 (57.9%)	64/113 (56.7%)
History of CABG	75/340 (22.1%)	25/113 (22.1%)
BNP (ng/l) (mean ± SD)	5118 ± 9693	7384 ± 9682
Primary mitral regurgitation	170/340 (50%)	8/113 (7.1%)
Secondary mitral regurgitation	68/340 (20%)	46/113 (40.7%)
Mixed aetiology	102/340 (30%)	59/113 (52.2%)

### Characteristics and procedural outcome of patients with severely depressed LV systolic function

**Figure 1:**
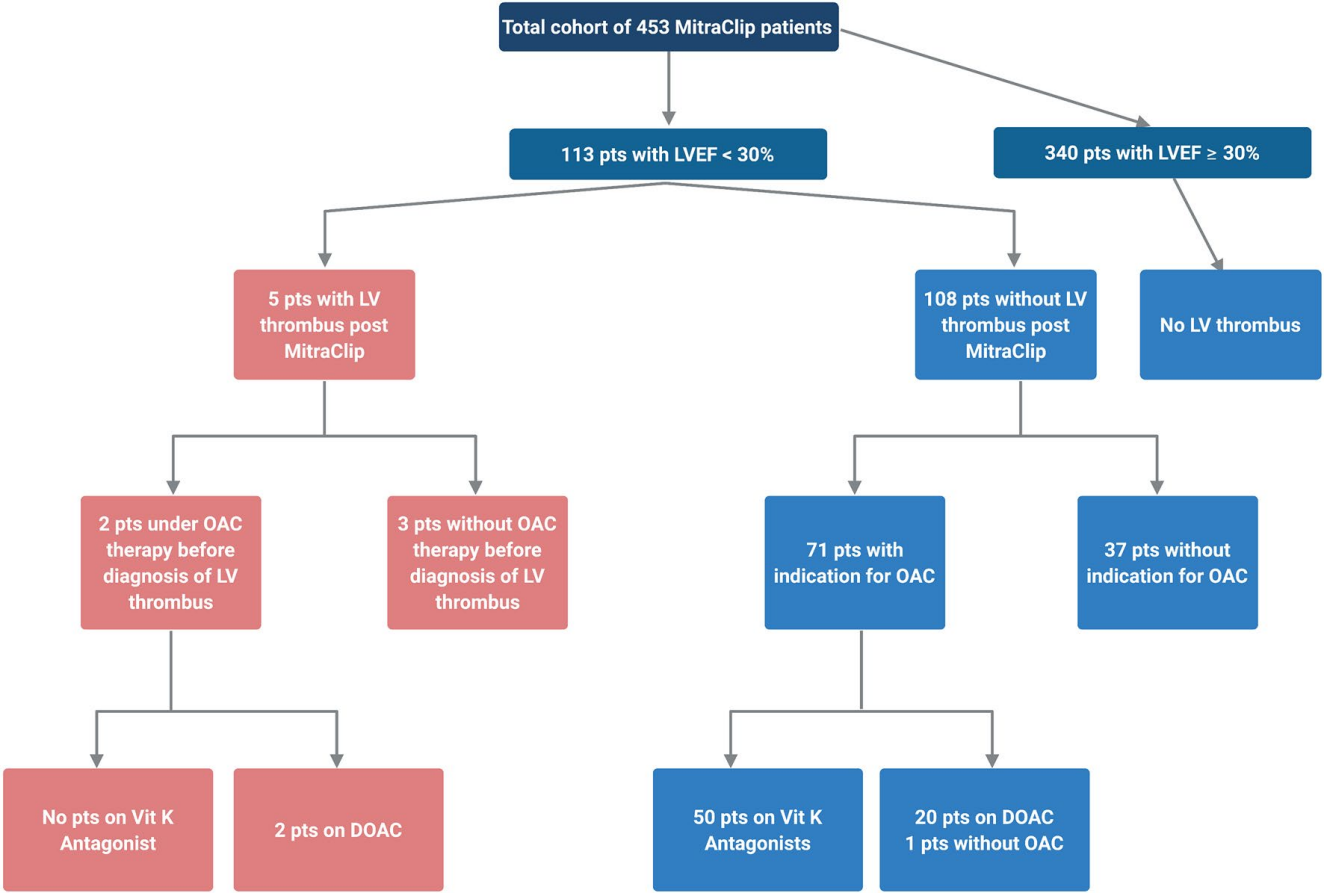
Prevalence of LV thrombus and anticoagulation therapy in total cohort.

As we found LV thrombi only in patients with LV-EF < 30%, this subgroup was subjected to further analyses (Fig. 1). The group consisted of 113 patients (24.9% of the total cohort). Baseline characteristics of the five patients diagnosed with LV thrombus as well as reference values of the cohort of patients with LV-EF < 30% are shown in Table 3. The cohort represents a typical real-world MitraClip cohort with advanced age and various comorbidities. All patients had left ventricular dilatation and a pathological sphericity index as well as severe mitral regurgitation before the MitraClip procedure (Table 4). Accordingly, levels of natriuretic peptides were high and invasively measured cardiac index was low. Etiology of heart failure was predominantly ischemic cardiomyopathy in two thirds of patients. Cardiovascular comorbidities such as diabetes, coronary heart disease and arterial hypertension were abundant in the cohort but less frequent in LV thrombus patients. No patient experienced LV thrombus formation before and pre-procedural echocardiograms did not show thrombus. Improvement of mitral regurgitation with one to three MitraClips was achieved in all patients that later developed LV thrombus. Trans-valvular gradient was high in only one patient (patient 3, mitral valve mean pressure gradient 8 mmHg), whereas mitral valve opening area was not impaired (mitral valve area 1.72 cm^2^, Table 4). Thus, we did not identify mitral valve valve stenosis following TMVR as a facilitator of LV thrombus formation.

**Table 3:** Baseline characteristics of the patients diagnosed with LV thrombus and the cohort with severely impaired left ventricular systolic function.

Baseline characteristics	Patient 1	Patient 2	Patient 3	Patient 4	Patient 5	MC/HFrEF population (LVEF > 30%) ± STD
Age	73	62	79	60	57	79
BMI	24.2	30.1	20.7	23.1	24.0	25.7 ± 4.9
ICM	Yes	No	Yes	Yes	No	40/69
DCM	No	Yes	No	No	Yes	29/69
GFR (ml/min)						50.4 ± 22
COPD	No	No	No	No	No	7/69
History of stroke	No	No	No	No	No	13/69
Diabetes mellitus	No	No	Yes	No	No	25/69
Art. hypertension	No	Yes	Yes	No	No	47/69
Coronary vessel disease	Yes	No	Yes	Yes	Yes	40/69
CHA_2_DS_2_-Vasc SCore	2	2	6	3	2	
BNP (ng/l)	3977	–	2097	5505	5684	7782 ± 9762
Indication for anticoagulation before LV thrombus diagnosis	No	Yes	No	No	Yes	
Oral anticoagulation	–	Rivaroxaban	No	–	Apixaban	
Indication	–	Varikosis	–	–	Afib	
History of TE	No	No	No	–	Yes	
History of cancer	Yes	No	Yes	No	No	

*Afib* atrial fibrillation, *BMI*– body mass index, *ICM* ischemic cardiomyopathy, *DCM* dilatative cardiomyopathy, *COPD* chronic obstructive pulmonary disease, *GFR* glomerular filtration rate, *MC* MitraClip.

**Table 4:** Echocardiographic and procedural data.

Echo/procedural parameters	Patient 1	Patient 2	Patient 3	Patient 4	Patient 5
LVEDD (mm)	74	58	62	70	83
LV ejection fraction (%)	25	29	16	19	18
MV area (cm^2^)	1.91	1.95	1.74	1.76	1.98
MR grade baseline	3	4	4	4	4
MR grade discharge	2	0	1	2	1
Clip no	3	1	3	2	2
Mean MV gradient (mmHg) post clip	2	5	8	3	1
Sphericity index	1.33	1.51	1.44	1.45	1.33
CI (l/m^2^/min)	1.4	n.d	1.6	1.8	1.1

*LVEDD* left ventricular enddiastolic diameter, *MV* mitral valve, *MR* mitral regurgitation.

### Role of background oral anticoagulation therapy

**Figure 2:**
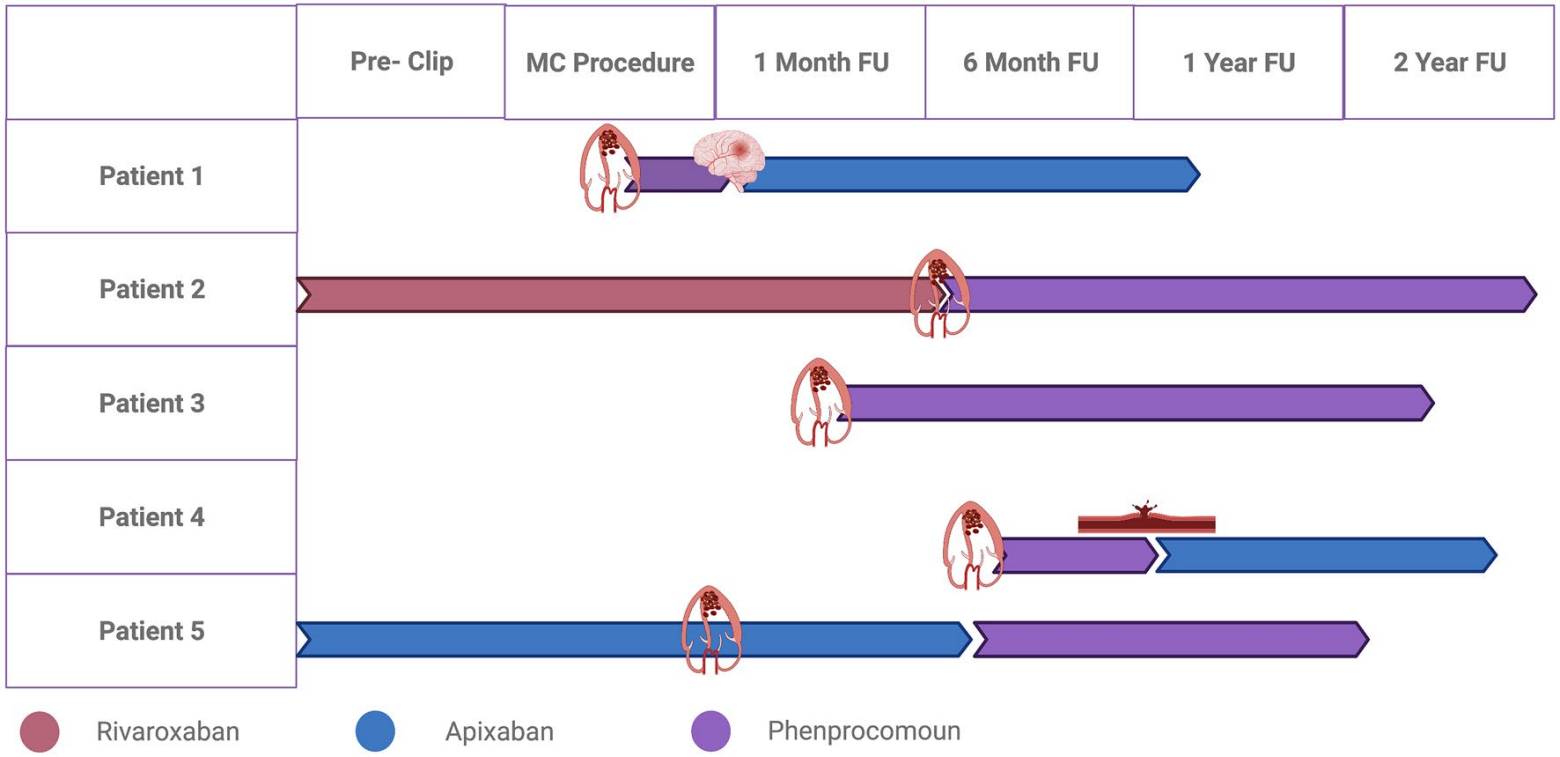
Gantt chart with time points of thrombus detection and prescribed anticoagulation therapy.

In the subgroup of patients with LV-EF < 30% that did not develop LV thrombus (n = 108), a total of 70 patients (64.8%) received oral anticoagulation therapy. Among these, the majority (32/71pts; 71.4%) was treated with a Vitamin K antagonist, whereas 20 patients were on a DOAC. One patient with an indication for oral anticoagulation did not take any anticoagulation therapy at all due to a history of bleeding. Importantly, two out of the five patients with thrombus diagnosis were under oral anticoagulation therapy with a DOAC, either Apixaban (due to atrial fibrillation with prior thromboembolism (splenic infarction)) or Rivaroxaban (history of deep venous thrombosis). Two patients had a history of cancer but were in remission without evidence of active disease, so that there was no indication for continuation of oral anticoagulation therapy (Table 3) (glomus cancer and prostate cancer, respectively). No patient was on therapy with a Vitamin K antagonist at the time of LV thrombus diagnosis. All thrombi were diagnosed post MitraClip procedure by transthoracic echocardiography. In one case, thrombus formation was diagnosed in the pre-discharge echocardiography, one thrombus was found at 1 month follow-up examination and in three cases the thrombus was diagnosed five months post MitraClip procedure (Fig. 2). After diagnosis of LV thrombus, all patients were treated with Phenprocoumon with a targeted international normalized ratio (INR) of 2.0–3.0. Two patients experienced bleeding events, both under bridging therapy with heparin. Patient one experienced secondary intracranial bleeding after stroke of suspected thromboembolic origin under bridging therapy without any severe sequelae. Patient four experienced bleeding into the CRT-D pocket under perioperative heparin bridging therapy. Both patients were switched to Apixaban following the bleeding event.

## Discussion

Left ventricular thrombus formation following the MitraClip procedure is a rare complication and was clearly associated with severely reduced left ventricular systolic function in our study. In the subgroup with LV-EF < 30%, five patients were identified (5/113, 4.4%) as being newly diagnosed with LV thrombus 1–5 months after the procedure. While the prevalence of left ventricular thrombus formation in patients with reduced left ventricular function is controversial in older reports with a small sample size^1–3^, a recently published very large retrospective study reported that LV thrombus occurred in 1.3% of patients with HFrEF (Zhou et al., ESC Heart Fail 2021, PMID: 33496071)^12^. The numbers we found (LV thrombus in 4.4% of patients with severely depressed LV-EF) are higher, therefore suggesting that mitral valve edge-to-edge repair increases the already increased risk of LV thrombus formation in this cohort.

We observed two cases of thrombus formation under direct oral anticoagulation (DOAC) therapy with Rivaroxaban or Apixaban, respectively. Whereas the exact reasons for thrombus development under DOAC therapy are unknown, effectivity of DOACs may be reduced in these patients with multimorbidity, as factors such as impaired renal function and altered body mass index may interfere with optimal effectivity of the drug. These cases led us to change the standard operating procedure at our center in a way that in patients with an indication for oral anticoagulation and with LV-EF < 30%, Vitamin K antagonists are routinely used following MitraClip, even when DOAC therapy was administered before. This change of strategy has drawbacks as several DOAC approval studies in atrial fibrillation showed significant advantages in terms of bleeding rates in comparison to Warfarin^13^. Even in patients without transcatheter mitral valve repair (TMVR) and LV thrombus, the ideal therapeutic strategy is unknown as contradicting studies have been published recently^5,14–16^. In our cohort, two bleeding events were observed under Vitamin K therapy. However, both of them occurred during the bridging of Vitamin K antagonists with heparin. This observation once more demonstrates that bridging dramatically increases the risk of bleeding, especially in patients with risk factors. Therefore, bridging therapy should only be applied in selected cases^17^. We believe that due to the evolvement of LV thrombi despite ongoing DOAC treatment and the high inherent thromboembolic risk of this diagnosis, the switch to a Vitamin K antagonist should be strongly considered after TMVR. Importantly, the INR needs to be closely monitored, and heparin bridging (e.g., in case of planned surgery) should be applied very cautiously, if at all.

In our cohort, only one patient (patient 2) would have met eligibility criteria for the COAPT trial^7^—most of the left ventricles would have been too large (LVEDD > 70 mm) or ejection fraction would have been too low (LVEF < 20%). However, this observation is in line with published real-world data that suggest that patients with similar echocardiographic characteristics are frequently treated with TMVR^18^, most probably, as up to date there are no echocardiographic parameters that clearly predict outcome^19^. An earlier publication of another large scale MitraClip Center in Munich, Germany, supports our findings. Orban et al.^10^ investigated a MitraClip Cohort in an earlier phase with a total of 150 patients treated between 06/2009 and 07/2012. They found a total of three patients with thrombus formation following MitraClip—all of these patients had dilated left ventricles and heart failure with reduced ejection fraction (LV-EF < 20%). Still, a number of differences between our study and the one by Orban et al. remain. These include the extent of LV function deterioration (≤ 20% vs. < 30%), the timing of thrombus diagnosis, baseline anticoagulation regime, and, clinical status of patients. All LV thrombi in Orban et al. were diagnosed in the very early post procedural phase (discharge echocardiography) whereas in our cohort it took up to five months until LV thrombus was found. Given this finding, the previous study may have missed LV thrombus due to the short follow-up. Furthermore, two out of three patients were on anticoagulation with Vitamin K antagonists (Phenprocoumon) before MitraClip but had insufficient INR values and PTT < 60 s in the post procedural phase. Indication for anticoagulation in patient one was a calcified structure in the LV wall before procedure. As calcified structures within the LV are mostly organized thrombi, this raises the question if the periprocedural anticoagulation regime with discontinuation of the Vitamin K antagonist may have led to the formation of appositional thrombus formation. The second patient was on oral anticoagulation due to atrial fibrillation and was in critical clinical condition because of cardiogenic shock prior and during the MitraClip procedure. All three patients died 1 week—3 months post procedure (2 pts due to heart failure 1 pt with pneumonia), indicating the very severe end stage heart failure. Thus, two out of three patients that developed LV thrombus in the previous study are not representative for the majority of patients treated with the MitraClip in Germany and Europe^20,21^ and interpretation and generalization of the study results is difficult. Thus, our data for the first time shed light on this important clinical szenario.

The five patients that developed an LV thrombus following MitraClip procedure did not differ significantly from patients without LV thrombus with respect to baseline characteristics or to echocardiographic parameters prior or following treatment. Thus, we were not able to identify factors that are associate with increased risk of thrombus formation after TMVR. Therefore, cardiologists that perform follow-up echocardiography after TMVR need to be especially alert and need to specifically rule out apical LV thrombus in patients with severely depressed LV systolic function. Hemodynamically and mechanistically, the alteration of LV inflow after MitraClip implantation via “split inflow”^9^ or spreading and deceleration of inflow in the setting of multiple devices in association with an already disease-modified LV geometry and LV function may critically impact on LV thrombus formation. But not only LV thrombus formation may appear following TMVR. There are published cases showing LA thrombus formation following TMVR^22,23^. These findings may also be linked to the change of flow by creating a double or triple transmitral inflow as the pendulum volume that is caused by relevant mitral regurgitations is dramatically reduced after successful TMVR. These hemodynamic considerations appear plausible, However, we have to clearly state the hypothetical nature of this notion.

Our study has several limitations due to its retrospective design, due to the generally low frequency of complications and the nature of detection of LV-thrombi by echocardiography that can be challenging. However, our study demonstrates 3 important aspects that are important for all cardiologists that follow up patients treated with the MitraClip. These include (1) the observation that LV thrombi may develop after MitraClip even under DOAC therapy (2) patients with severely depressed LV-EF are at risk (3) heparin bridging of Vitamin K antagonists in this cohort of patients is associated with a high risk of bleeding.

## Conclusion

LV thrombus formation following mitral valve edge-to-edge repair implantation is a rare complication that occurs exclusively in patients with severely depressed LV-EF even under DOAC therapy. LV thrombus may have severe impact on patient outcomes and quality of life. Treatment with Vitamin K antagonists should be considered in this subgroup if an indication for oral anticoagulation exists.

### Impact on daily practice

In patients with heart failure with severely reduced LV systolic function treated with TMVR, increased awareness for LV thrombus formation in routinely performed transthoracic echocardiography studies is warranted. In patients with an indication for anticoagulation following TMVR, anticoagulation therapy with Vitamin K antagonists should be considered.

## Data Availability

The datasets generated during and analysed during the current study are available from the corresponding author Henrik ten Freyhaus on reasonable request.

## Abbreviations

Afib: Atrial fibrillation
BNP: Brain natiuretic peptide
COPD: Chronic obstructive pulmonary disease
DAPT: Dual antiplatelet therapy
DCM: Dilatative cardiomyopathy
DOAC: Direct oral anticoagulant
GFR: Glomerular filtration rate
HFrEF: Heart failure with reduced ejection fraction
ICM: Ischemic cardiomyopathy
LV: Left ventricle
LV-EF: Left ventricle ejection fraction
OAC: Oral anticoagulation therapy
TMVR: Transcatheter mitral valve repair
TTE: Transthoracic echocardiography
